# The Role of Adjuvant Therapy for the Treatment of Micrometastases in Endometrial Cancer: A Systematic Review and Meta-Analysis

**DOI:** 10.3390/jcm13051496

**Published:** 2024-03-05

**Authors:** Carlo Ronsini, Stefania Napolitano, Irene Iavarone, Pietro Fumiento, Maria Giovanna Vastarella, Antonella Reino, Rossella Molitierno, Lugi Cobellis, Pasquale De Franciscis, Stefano Cianci

**Affiliations:** 1 Department of Woman, Child and General and Specialized Surgery, University of Campania “Luigi Vanvitelli”, 80138 Naples, Italy; ireneiavarone2@gmail.com (I.I.); pietro.fumiento@virgilio.it (P.F.); mariagiovanna.vastarella@unicampania.it (M.G.V.); antonella.reino.11@gmail.com (A.R.); molitiernorossella@gmail.com (R.M.); luigi.cobellis@unicampania.it (L.C.); pasquale.defranciscis@unicampania.it (P.D.F.); 2 Division of Medical Oncology, Department of Precision Medicine, University of Campania “Luigi Vanvitelli”, 80138 Naples, Italy; stefania.napolitano@unicampania.it; 3 Gynecologic Oncology Unit, Women Wealth Area, Department of Woman and Child Health and Public Health, Fondazione Policlinico Universitario A. Gemelli IRCCS, Università Cattolica del Sacro Cuore, 00136 Rome, Italy

**Keywords:** endometrial cancer, micrometastases, radiation therapy, chemotherapy, adjuvant

## Abstract

Endometrial cancer is the most incident gynecological cancer. Lymph node dissemination is one of the most important factors for the patient’s prognosis. Pelvic lymph nodes are the primary site of extra-uterine dissemination in endometrial cancer (EC), setting the 5-year survival to 44–52%. It is standard practice for radiation therapy (RT) and/or chemotherapy (CTX) to be given as adjuvant treatments to prevent the progression of micrometastases. Also, administration of EC patients with RT and/or CTX regimens before surgery may decrease micrometastases, hence the need for lymphadenectomy. The primary aim of the systematic review and meta-analysis is to assess whether adjuvant RT and/or CTX improve oncological outcomes through the management of micrometastases and nodal recurrence. We performed systematic research using the string “Endometrial Neoplasms” [Mesh] AND “Lymphatic Metastasis/therapy” [Mesh]. The methods for this study were specified a priori based on the recommendations in the Preferred Reporting Items for Systematic Reviews and Meta-Analyses (PRISMA) statement. Outcomes were 5-year overall survival, progression-free survival, recurrence rate, and complications rate. We assessed the quality of studies using the Newcastle–Ottawa Scale (NOS). A total of 1682 patients with stage I-to-IV EC were included. Adjuvant treatment protocols involved external-beam RT, brachytherapy, and CTX either alone or in combination. The no-treatment group showed a non-statistically significant higher recurrence risk than any adjuvant treatment group (OR 1.39 [95% CI 0.68–2.85] *p* = 0.36). The no-treatment group documented a non-statistically significant higher risk of death than those who underwent any adjuvant treatment (RR 1.47 [95% CI 0.44–4.89] *p* = 0.53; I2 = 55% *p* = 0.000001). Despite the fact that early-stage EC may show micrometastases, adjuvant treatment is not significantly associated with better survival outcomes, and the combination of EBRT and CTX is the most valid option in the early stages.

## 1. Introduction

Endometrial cancer (EC) is the most diffuse gynecological neoplasia worldwide, occurring in 2–3% of patients [1]. The main risk factor for endometrial cancer is exposure to an excessive estrogenic environment that is not balanced by an adequate amount of progesterone; therefore, obese patients are more at risk due to the production of estrogen by aromatase in adipose tissue, patients affected by PCOS due to chronic anovularity with deficiency in progesterone production with relative hyperestrogenism, patients suffering from diabetes mellitus and arterial hypertension, early menarche, late menopause, nulliparity, patients being treated with tamoxifen, and patients after the fourth and fifth decade of life [1,2]. Vaginal bleeding is the most common sign of endometrial malignancy, and thanks to it, more than 75% of women are diagnosed with early-stage disease. Following abnormal uterine bleeding, especially in menopausal women, it would be necessary to carry out a transvaginal pelvic ultrasound, which will detect endometrial thickening (where endometrial thickening in a menopausal woman means a thickness greater than 4 mm). From here, it would be necessary to carry out a diagnostic hysteroscopy with targeted biopsy near the areas suspected of malignancy (for example, areas with vascular atypia). Histological examination will give definitive confirmation.

There is also a family predisposition to develop endometrial carcinoma. Among them, we remember Lynch syndrome type II, in which there is a greatly increased risk of developing malignant tumors of the colon, endometrium, and ovary. Therefore, it is of fundamental importance to search for hereditary tumors when a young patient with endometrial cancer is found.

This leads to a favorable prognosis with an overall 80–85% survival at 5 years [2,3]. Only 10% of patients with clinical early-stage EC have lymph node involvement [4,5]. Otherwise, it would be appropriate to establish standard treatment protocols to appraise the risk of relapse and the adverse effects of unnecessary treatments [6,7,8,9]. Nowadays, the main issue regards the extent of surgical dissection—also for staging purposes—especially for lymph nodes [2,6,7]. Macrometastases are defined as those with dimensions >2 mm; micrometastases are defined as those with dimensions between 0.2 and 2 mm or greater than 200 cells; isolated tumor cells are released if <0.2 mm or <200 cells [6].

The presence of micrometastases into the pelvic lymph nodes—when EC is limited to the uterine corpus—is estimated to be 5%-to-18% [2]. The involvement of retroperitoneal lymph nodes—embracing pelvic and/or para-aortic nodes—shows worse prognosis, with 44%–to-52% overall survival (OS) at 5 years [2]. The standard treatment for EC confined to the uterus is class A radical hysterectomy—according to the Querleu–Morrow classification—with bilateral salpingo-ooforectomy [2,10,11]. Pelvic lymph nodes are the main site of extra-uterine dissemination of the disease, setting the prognosis to 44–52% at 5 years [2]. Indeed, lymphadenectomy may be recommended for all EC stages, whereas some may recommend selective pelvic and para-aortic lymph node dissection based on tumor grading and myometrial invasion [5]. The extension of the surgical intervention suggests that the incidence of regional lymph node involvement in EC could be 10%-to-34% in high-risk women but up to 5% in low-risk categories [5]. Internationally accepted clinical and pathological risk factors estimating prognosis are lymph node involvement, lymphovascular space invasion (LSVI), tumor grading, patient age, and T classification [12,13]. Data from PORTEC 1 and 2 trials also recognized LVSI as a risk factor for pelvic nodal relapse [14]. LVSI is represented by neoplastic cells entering uterine lymphatic or vascular spaces and occurs in 15% of stage I-II EC [15,16]. It is standard practice for radiation therapy (RT) and/or chemotherapy (CTX) to be given as adjuvant treatments. Administration of EC patients with radiation therapy (RT) and/or chemotherapy (CTX) regimens—before surgery also—may decrease the rate of micrometastases, hence the need for lymphadenectomy [17,18]. Nowadays, sentinel lymph node (SLN) mapping is considered a valid alternative to systematic lymphadenectomy in order to assess nodal involvement [19,20,21]. The sentinel lymph node technique consists of identifying the first drainage lymph node station of the uterus through an endocervical injection of indocyanine green, which thus allows us to easily identify the sentinel lymph node and then remove and analyze it. In some cases, this technique fails, and therefore a systematic pelvic lymphadenectomy, which is certainly a more invasive procedure, is necessary for staging.

The potential management of micrometastases remains unclear. In addition, SLNs may be evaluated for pathologic ultrastaging because it provides prognostic details [14,15,19,20].

The primary objective of the present systematic review and meta-analysis is to assess whether adjuvant RT and/or CTX regimens improve oncological outcomes through the management of micrometastases and lymph node relapse. Secondarily, we aim to determine whether there is a suitable and optimal combination of those treatment regimens for different categories of patients.

## 2. Materials and Methods

The present study was exempted from ethical approval because it does not include interventions on human subjects. The methods for this study were specified a priori based on the recommendations in the Preferred Reporting Items for Systematic Reviews and Meta-Analyses (PRISMA) statement [22]. The review is registered on PROSPERO as ID431489.

### 2.1. Search Method

We performed systematic research for records about the eventual use of different therapeutic regimens in managing micrometastases in EC via PubMed, EMBASE, Scopus, Google Scholar, Clinical-trials.gov, and the Cochrane Central Register of Controlled Trials in April 2023. We made no restriction on country or year of publication and considered only English-entirely published studies. We adopted the following string of idioms in each database to identify studies fitting to our review’s topic: “Endometrial Neoplasms” [Mesh] AND “Lymphatic Metastasis/therapy” [Mesh].

### 2.2. Study Selection

**Figure 1:**
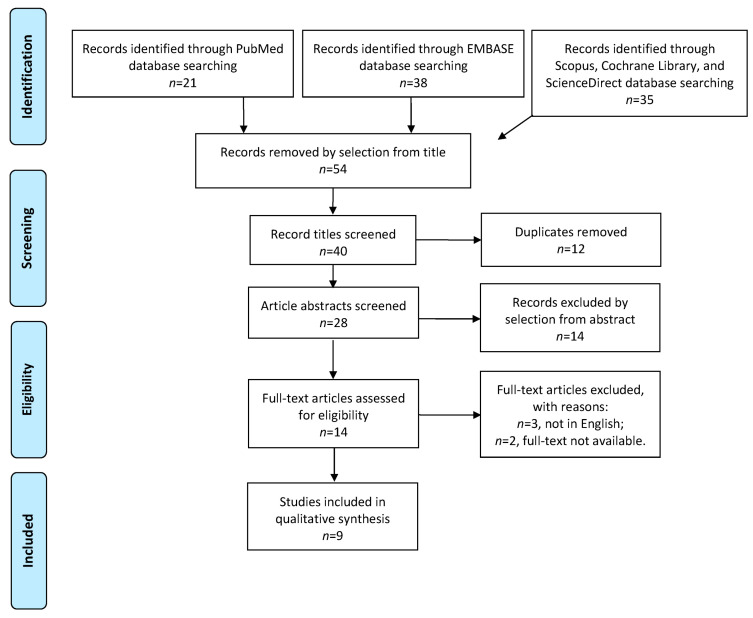
Preferred Reporting Items for Systematic Reviews and Meta-Analyses (PRISMA) flowchart.

Study selection was made independently by I.I. and P.F. In cases of discrepancy, C.R. decided on inclusion or exclusion. Our population included patients with histological diagnoses of EC and lymph node involvement. No restrictions concerning FIGO stage, tumor grading, or the site of lymph node recurrence were applied. Recurrence and survival outcomes were analyzed, and treatment options—RT, CTX, and HT—were compared. The inclusion criteria were the following: (1) studies that included patients histologically diagnosed with EC with microscopic involvement of at least one lymph node (<2 mm); (2) studies reporting at least one outcome of interest: OS, recurrence rate (RR); progression-free survival (PFS) after either observation or adjuvant treatment of micrometastases; and (3) peer-reviewed articles, published originally. We excluded the following: non-original studies, pre-clinical trials, animal trials, abstract-only publications, and articles in a language other than English. If possible, the authors of studies that were published as conference abstracts were contacted via e-mail and asked to provide their data. We mentioned the studies selected and all the reasons for exclusion in the Preferred Reporting Items for Systematic Reviews and Meta-Analyses (PRISMA) flowchart (Figure 1). We assessed all the included studies concerning potential conflicts of interest.

### 2.3. Statistical Analysis

Heterogeneity among comparative studies was tested using the Chi-square test and I-square tests [23]. Risk rates and 95% confidence intervals (CI) were used for dichotomous variables. Statistical analysis was conducted using fixed-effect models without significant heterogeneity (I2 < 50%) or using random-effect models if I2 > 50%. The 5-year OS, 5-year PFS, 5-year RR, and complications rate were the clinical outcomes calculated in each study as percentages. The chi-square test was used to compare continuous variables. Review Manager version 5.4.1 (RevMan 5.4.1) and IBM Statistical Package for Social Science (IBM SPSS version 25.0) for MAC were used for statistic calculation. For all the performed analyses, a *p*-value <0.05 was considered significant.

### 2.4. Quality Assessment

We assessed the quality of the included studies using the Newcastle–Ottawa Scale (NOS) [24]. That assessment scale uses three broad factors (selection, comparability, exposure), with scores ranging from 0 (lowest quality) to 9 (best quality). Two authors (I.I. and P.F.) independently rated the studies’ quality. Any disagreement was resolved by discussion or consultation with C.R. We have reported the NOS scale in Appendix A. We used a funnel plot analysis to establish publication bias.

## 3. Results

### 3.1. Studies’ Characteristics

After the database search, 40 articles matched the search criteria. After removing records with no full-text, duplicates, and wrong study designs (e.g., reviews), 14 were eligible. Nine articles matched the inclusion criteria and were included in the systematic review. Four articles were non-comparative, single-armed studies evaluating only adjuvant treatment regimens for micrometastases [25,26,27,28]. The other five were comparative studies between observation and adjuvant treatments for micrometastases and were included in quantitative analysis (Figure 1) [29,30,31,32,33]. The countries where the studies were conducted, the publication year range, the studies’ design, the FIGO (International Federation of Gynecology and Obstetrics) stage of disease, the number of participants, and adjuvant treatment protocols are summarized in Table 1. The quality of all studies was assessed by NOS [24] (Appendix A). Overall, the publication years ranged from 2014 to 2021 [25,26,27,28,29,30,31,32,33]. In total, 1682 patients with EC were included [25,26,27,28,29,30,31,32,33]. The follow-up (FU) period ranged from 17.8 to 84.8 months on average [25,26,27,28,29,30,31,32,33].

### 3.2. Outcomes

A total of 1682 patients with stage I-to-IV EC were included in the review [25,26,27,28,29,30,31,32,33]. Adjuvant treatment protocols included external beam (EB) RT, BT, and CTX either alone or in combination [25,26,27,28,29,30,31,32,33]. The studies by Raimond et al. and Backes et al. presented the highest PFS rates at 5 years (91% and 95%, respectively), together with the lowest 5-year RR (9% and 5.1%, respectively) [25,33]. Those articles showed data neither about OS nor about the adverse effects of adjuvant therapy [25,33]. In both cohorts, patients were at an early stage of the disease, and adjuvant treatment protocols for pelvic nodal relapse included EBRT or BT [25,33]. Backes et al. used EBRT ± CTX in order to treat the para-aortic site of recurrence as well [33]. The Forsse et al. cohort included patients with FIGO I-to-IV stages of the disease, and nodal relapse was located on pelvic and para-aortic lymph nodes [28]. Patients were administered EBRT ± CTX, BT, or HT [28]. The 5-year PFS and OS rates were 81.5% and 75%, respectively, whereas the 5-year RR was 17% [28]. No data were shown regarding iatrogenic toxicities [28]. In the study by Piedimonte et al., nodal recurrence was located on the iliac, obturator, and para-aortic lymph nodes [27]. Patients at FIGO stage I were administered EBRT ± CTX, and the 5-year PFS, OS, and RR were 77.5%, 50%, and 8.7%, respectively [27]. Adverse events were 9.1%, according to the National Cancer Institute Common Toxicity Criteria ≥ 3. In addition, Onal et al. described recurrence in pelvic and para-aortic lymph nodes [30]. Although PFS and OS at 5 years were 77% and 71%, respectively, the 5-year RR was 34.7%. In that case, patients were administered EBRT ± CTX, and the FIGO stage of the disease was IIIC1 [30]. Lee et al. presented the lowest 5-year PFS and OS rates (51% and 48%, respectively) [26]. Women with para-aortic lymph node recurrence were administered EBRT ± CTX, and the FIGO stage of the disease was IIIC1 [26]. Those results are summarized in Table 2.

### 3.3. Meta-Analysis

The five studies comparing no treatment for micrometastases with any other adjuvant treatment were enrolled in the meta-analysis. A total of 986 patients were analyzed. A total of 300 patients in the no-treatment arm were compared with 686 patients who underwent any adjuvant treatment, as described in Table 1, by exploring the DFS outcome. Because of the high heterogeneity (I2 > 50%; *p* < 0.00001), a random-effects model was applied.

**Figure 2:**
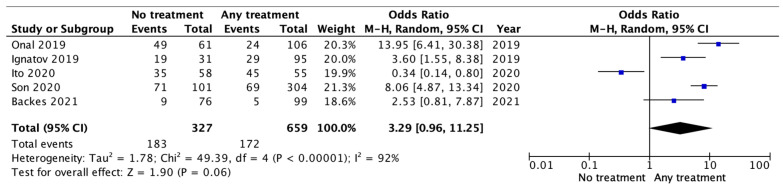
Risk of recurrence.

The no-treatment group showed a non-statistically significant higher recurrence risk than any adjuvant treatment group (OR 1.39 [95% CI 0.68–2.85] *p* = 0.36), as shown in Figure 2.

**Figure 3:**
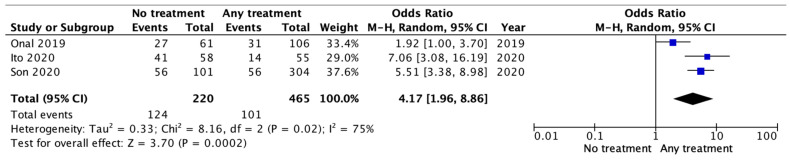
Risk of death.

We performed a sub-analysis for the patients looking at the risk of death. Unfortunately, only three of the five comparative studies reported useful data: 193 patients for the no-treatment group and 492 for any other adjuvant treatment group. In addition, in that analysis, the no-treatment group documented a non-statistically significant higher risk of death than those who underwent any adjuvant treatment (RR 1.47 [95% CI 0.44–4.89] *p* = 0.53; I2 = 55% *p* = 0.000001), as documented in Figure 3.

## 4. Discussion

The cohorts analyzed in our study were principally administered with a combination of EBRT and CTX [25,26,27,30,33]. Raimond et al. and Backes et al. documented the highest PFS rates (91% and 95%, respectively) and the lowest RR (9% and 5.1%, respectively) [25,33]. In particular, the Raimond et al. cohort included patients with FIGO stage I only, whereas in the Backes et al. study, patients were FIGO stage I-II [25,33]. Those data demonstrate that EBRT ± CTX may be a valid adjuvant treatment option for patients with early-stage EC and micrometastases in pelvic and para-aortic lymph nodes. In parallel, EBRT ± CTX showed lower survival outcomes in the Lee et al. study [26]. In that case, patients had FIGO IIIC2 EC and metastases in para-aortic nodes, revealing that adjuvant therapy may not be sufficient in advanced stages of disease [26]. The diagnosis of micrometastases in women with apparent node-negative EC could be crucial for prognosis [34]. In fact, the presence of isolated tumor cells (ITCs) and nodal metastases is related to an increased number of breast, colon, and prostate cancers [35,36,37,38,39]. Results regarding SLN pathologic ultrastaging confirm that women with ITCs may be managed as node-negative [40]. In that specific context, adjuvant treatment can be assessed according to uterine risk factors. Whereas women with micrometastases can be managed as node-positive [40]. Based on our study, due to the heterogeneity of the data and the exiguous number of fitting records, it is impossible to determine the correct sequence of RT and CTX [25,26,27,30,40]. In order to avoid that bias, the extent of the sample size for each category of treatment could allow authors to perform further trials to assess recurrence and survival outcomes after the use of RT, CTX, and combinations of those regimens, respectively. However, the molecular profile of EC patients should be assessed when considering adjuvant treatment regimens [40]. For example, there is evidence that cytokeratin expression in regional lymph nodes is related to the positivity of LVSI (*p* = 0.011), deep myometrial invasion, and disease recurrence (*p* < 0.0001) [41,42]. In addition, the expression of cytokeratin in metastatic lymph nodes did not concern tumor cells, and it was related to disease recurrence in early-stage EC (*p* = 0.0096) [41]. Recent evidence demonstrated that the molecular subtype was significantly related to nodal metastases (*p* = 0.004) [43]. In particular, the expression of p53abn presents nodal involvement in 44.8% of patients; POLEmut shows nodal metastases in 14.2% of cases; MMRd in 14.9%; and NSMP in 10.8% [43]. Regarding metastases’ entity, subsequent evidence compared oncological outcomes in women with ITCs, micrometastases, and macrometastases [44,45,46]. Women with micrometastases showed significantly better survival outcomes compared to women with macrometastases, and the difference between micrometastases and negative lymph nodes in PFS was not significant, demonstrating that negative lymph nodes are not a better prognostic factor compared to micrometastases [44,45,46]. In the García Pineda et al. analysis, the authors provide information regarding the histopathological features of EC patients with micrometastases [46]. For example, most micrometastases showed endometrioid histology and low grading, and no difference between patients with and without nodal metastases was noticed [46]. Instead, myometrial invasion appeared as an independent risk factor for nodal metastases [46]. The strength of our analysis is that it embraces all studies assessing whether adjuvant EBRT and/or CTX regimens improve oncological outcomes through the management of micrometastases and lymph node relapse. One limitation of our study is the exiguous number of available records in the scientific literature. Another point of weakness is the heterogeneity of the data; in fact, patients are not stratified according to either the FIGO stage of the disease or the site of micrometastases. Moreover, most studies included in our systematic review did not specifically distinguish their patients in EBRT + CTX and EBRT without CTX arms, jeopardizing the consistency of their analyses [26,27,28,30,32]. To date, our meta-analysis documents that there is no consistent benefit from adjuvant treatment. It could depend on the lower risk of positive lymph nodes in patients with micrometastases—especially for ITCs—compared to macrometastases. In parallel, recent evidence shows that patients with ITCs in SLNs could not benefit from adjuvant treatment [33]. In parallel, an issue concerning distant sites of EC dissemination is loco-regional recurrence [47]. It could be attributed to molecular pathways of tumor instability [48,49,50]. The tumor microenvironment, involving, for example, LVSI, shows a crucial role in EC relapse [51,52]. Moreover, the effectiveness of surgical staging may be confounding [53,54]. However, improvement of SLN mapping to the detriment of systematic lymphadenectomy can understage EC [55]. In our opinion, that issue partially justifies loco-regional recurrence and is connected to lymph nodes or distant relapse. In the case of fertility-sparing treatment (FST), grading mainly predicts the response to hormonal therapy (HT) [56]. In fact, Medroxyprogesterone Acetate (MPA) leads to a 37% response rate (RR) in well-differentiated EC, whereas it leads to a 9% RR in G3 neoplasm [57]. In addition, HT can be influenced by the expression of the cell adhesion molecule L1-CAM, which is prone to invasiveness, metastatization, and relapse [58]. Although early-stage ECs may present micrometastases, adjuvant treatment regimens are not significantly associated with better survival outcomes, and the combination of EBRT and CTX is the most valid option in the early stage of disease. Molecular biomarkers could predict the efficacy of FST on EC. For example, deep myometrial invasion (DMI) is not independent of The Cancer Genome Atlas (TCGA) in assessing OS in EC, but it influences RR [59]. Moreover, similarly to other conditions—such as endometriosis—new strategies may be applied to detect micrometastases through liquid biopsy [60]. This allows for identifying in the serum of patients with EC, or in different cellular compartments, specific micro-RNAs that could be used as biomarkers of the disease [60]. In addition, knowledge of lymph node status, even if microscopic, could help modulate surgical aggressiveness, minimizing the risks of iatrogenic comorbidities [61,62,63].

Similarly, microbial signatures may subtend a specific pattern of clinical and oncological outcomes in women with EC [64]. The above-mentioned molecular methods for outcome prediction—as the miRNAs and the microbiota—might also be taken into account in a risk prediction model, even though the data allowing for establishing an optimal distinction of risk groups are still extremely poor. There is little evidence of that issue in endometriosis-affected patients, and it would be appropriate to investigate whether a similar mechanism could distinguish EC. In the end, the presence of micrometastases is a risk factor that should be contextualized in the patient’s clinical presentation in order to optimize the personalization of treatment. Avoiding adjuvant treatments where they should not be necessary has clinical and sociological value. Conversely, the goal of chronicizing oncologic disease and minimizing the risk of recurrence is the moral duty of the clinician. The importance of detecting micrometastases is highlighted by the pathological ultrastaging obtained through SLN mapping [65]. The SLN mapping procedure is gaining consensus in EC staging because it avoids unnecessary surgery-related morbidity, preserving the advantages of surgical staging [65]. There is increasing evidence that SLNs with ICG during laparoscopy assures a safe and valid mapping compared to methylene blue and radiolabeled albumin nanocolloid [65]. This might be fundamental for preoperative imaging [65]. Moreover, ICG fluorescence shows efficiency in terms of timing and nodal detection rates [65].

Today, with the new molecular classification, the presence of POLmut (polymerase-mutated), MMRd (mismatch repair deficient), NSMP (non-specific molecular profile), and p53abn (p53 abnormal) takes on a key role from a prognostic point of view [66].

The present study has several limitations. First of all, the authors made a very limited stratification of patients according to the FIGO stages and the sites of micrometastases. Therefore, as difficult as it is to establish the exact weight exerted by micrometastases in the oncologic history of patients with CE, it turns out to be a clinical fact that should not be disregarded. Unfortunately, we do not have enough evidence supporting the role of a specific combination of adjuvant treatment regimens in the different histological subtypes of EC. Moreover, due to the paucity of data, it is impossible to assess the optimal strategy of combining RT and CTX in different categories of EC patients. Another limitation of the analysis lies in heterogeneity. In particular, further studies are needed to address or mitigate that bias. Heterogeneity would be diminished by the setup of prospective multicenter studies involving a wider sample size, to be stratified based on the FIGO stage, site of nodal recurrence, and longer FU time. Those issues may fill the gaps in knowledge and suggest potential directions for future investigations.

## 5. Conclusions

Despite the fact that early-stage EC may show micrometastases, especially in pelvic and para-aortic lymph nodes, the use of adjuvant treatment is not significantly associated with better survival outcomes. However, those data highlight the importance of targeted therapy conforming to the FIGO stage of the disease. The combination of EBRT and CTX is the most diffuse and valid option for early-stage EC patients, although further evidence is needed to optimize and standardize adjuvant treatment protocols based on SLN mapping. It would also be appropriate to perform further trials in order to analyze survival outcomes according to the stage of the disease and the site of recurrence and to perform a more detailed stratification of advanced-stage patients.

## Figures and Tables

**Table 1:** Characteristics of included studies.

Author, Year of Publication	Country	Period of Enrollment	Study Design	FIGO Stage	No. of Participants	Adjuvant Treatment	Median FU Period (Months)
Single-arm studies							
Raimond, 2014 [25]	France	2000–2012	Retrospective observational multi-center cohort study	I	156	EBRT, BT	N/A
Lee, 2017 [26]	USA	1990–2015	Perspective interventional mono-center cohort study	IIIC2	72	EBRT ± CTX	43.0
Piedimonte, 2018 [27]	Canada	2012–2018	Retrospective observational mono-center case-control study	I	23	EBRT + CTX	24.0
Forsse, 2020 [28]	Norway	2001–2019	Perspective interventional mono-center cohort study	I-IV	445	EBRT, BT, CTX ± RT, HT	49.0
Comparative studies—adjuvant treatment vs. LN-included for meta-analysis							
Ignatov, 2019 [29]	Germany	2000–2017	Prospective interventional multi-center case-control study	N/A	126	EBRT	84.8
Onal, 2019 [30]	Turkey	2000–2016	Retrospective observational mono-center case-control study	IIIC1	167	EBRT ± CTX	49.0
Ito, 2020 [31]	Japan	2008–2018	Retrospective observational multi-center case-control study	N/A	113	High-dose salvage RT	17.8
Son, 2020 [32]	USA	2005–2015	Retrospective observational multi-center case-control study	IA-IB-II	405	EBRT ± BT ± CTX, HT	69.0
Backes, 2021 [33]	USA	2005–2017	Retrospective observational multi-center cohort study	IA-IB-II	175	EBRT ± CTX, BT	31.0

FIGO: International Federation of Gynecology and Obstetrics; FU: follow-up; EBRT: external-beam radiation therapy; BT: brachytherapy; CTX: chemotherapy; HT: hormone therapy.

**Table 2:** Outcomes.

Author, Year of Publication	LNs	5-Year PFS (%)	5-Year OS (%)	5-Year RR (%)	National Cancer Institute Common Toxicity Criteria for Adverse Events ≥3 (%)
Single-arm studies					
Raimond, 2014 [25]	Pelvic	91.0	N/A	9	N/A
Lee, 2017 [26]	Para-aortic	51.0	48.0	51.4	0.0
Piedimonte, 2018 [27]	Iliac, obturator, para-aortic	77.5	50.0	8.7	9.1
Forsse, 2020 [28]	Pelvic, para-aortic	81.5	75.0	17.0	N/A
Comparative studies—adjuvant treatment vs. LN-included for meta-analysis					
Ignatov, 2019 [29]	Pelvic, para-aortic	70.0	N/A	30.0	N/A
Onal, 2019 [30]	Pelvic, para-aortic	77.0	71.0	34.7	N/A
Ito, 2020 [31]	Para-aortic, iliac, presacral, obturator	17.5	74.8	25.1	0.0
Son, 2020 [32]	Pelvic, para-aortic	77.2	81.5	18.0	N/A
Backes, 2021 [33]	Pelvic, para-aortic	95.0	N/A	5.1	N/A

LNs: lymph nodes; PFS: progression-free survival; OS: overall survival; RR: recurrence rate.

## Data Availability

Data supporting conclusions of the study can be found in the Referenct List.

## Appendix A

**Table A1:** Newcastle–Ottawa Scale.

Single-arm studies					
**Author, Year of Publication**	**Country**	**Selection**	**Comparability**	**Exposure**	**Total**
Raimond, 2014 [25]	France	3	2	2	7
Lee, 2017 [26]	USA	3	2	3	8
Piedimonte, 2018 [27]	Canada	3	2	3	8
Forsse, 2020 [28]	Norway	3	2	2	7
Comparative studies—adjuvant treatment vs. LN-included for meta-analysis					
Ignatov, 2019 [29]	Germany	3	3	2	8
Onal, 2019 [30]	Turkey	3	3	3	9
Ito, 2020 [31]	Japan	3	3	2	8
Son, 2020 [32]	USA	3	3	3	9
Backes, 2021 [33]	USA	3	3	3	9
